# Supplementary material for: Rift Valley fever knowledge, mitigation strategies and communication preferences among male and female livestock farmers in Eastern Province, Rwanda
Source: PLoS Negl Trop Dis. 2021 Aug 23;15(8):e0009705. doi: 10.1371/journal.pntd.0009705 (PMC8412303; doi:10.1371/journal.pntd.0009705)
Supplement: S1 RVF Survey Questionnaire — (DOC) [file pntd.0009705.s001.doc]

1) District

2) Sector

3) Cell

4) What is your gender?

5) How old are you (years)?

6) What is your level of education?

7) How long have you been raising livestock?

8) What types of animals do you own?

Answer options:

Goat

Cattle

Sheep

Chicken

Pig

Other

8a) If other, what types of animals?

9) Do you eliminate standing water sources and minimize flooding in your farmland?

9a) Why do you do this?

10) How do you minimize your contact with mosquitoes?

11) Do you think that anything about tending to your livestock poses a health risk to you?

11a) What are those risks?

12) Are you aware of any diseases that you might catch from contact with animals?

12a) Can you provide some diseases?

13) Are you aware of any diseases that animals might catch from contact with humans?

13a) Can you provide some diseases that can spread from humans to animals?

14) Have you heard about Rift Valley Fever?

14a) Who told you about RVF?

Options:

Another farmer

Community Health Worker

Community Member

Government Official

Veterinarian

Other

14b) If other, who told you?

15) Did you hear about RVF through any of the below channels (choose all that apply).

Options:

In a conversation with someone else

On the phone

On the radio

Newspapers

Internet

Educational Materials

Other

15a) If other, how did you hear about RVF?

16) Do you hear about disease outbreaks on the radio?

17) When you hear information on the radio, do you share it with your community members?

17a) How do you share the disease information?

18) What do you think is the best way to receive information?

Options:

Talking in person

Talking on the phone

Sending letters

Listening to the radio

Reading newspapers

Other

18a) If other, what do you think is the best way to receive information?

19) Do you know how livestock become infected with RVF?

19a) If yes, how do livestock become infected?

20) Can humans be infected with RVF?

20a) How do humans become infected with RVF?

21) Do you have special practices for protecting yourself while you are tending to your animals?

21a) If yes, what special practices do you use to protect yourself?

21b) If no, why don't you use protective equipment?

Options:

Too expensive

They are not available

No access to market

There are no risks so I don’t own any

Other

21c) If other, why don’t you use protective equipment?

22) Do you ever slaughter your own animals?

22a) If yes, what species do you slaughter?

Options:

Cattle

Goat

Sheep

Chicken

Pig

23) Do you wear personal protective equipment when slaughtering your own animals?

23a) If yes, what do you wear?

Options:

Gloves

Lab coat

Gum boots

Hat

23b) Why do you wear these things when slaughtering?

23c) If no, why don't you wear protective equipment while slaughtering?

24) Do you have soap and water available on your farm to wash your hands?

24a) If yes, when do you wash your hands as it relates to working with animals?

Options:

Before and after working with animals

Only after working with animals

Only before working with animals

Before and after preparing meat and animal organs/tissues

Only before preparing meat and animal organs/tissues

Only after preparing meat and animal organs/tissues

Other

24b) If other, when do you wash your hands?

25) What do you do when your animal is sick?

Options:

Report the disease

Call a veterinarian

Slaughter and eat the animal

Sell the animal

Treat it by yourself

25a) If you report the disease, how do you report it and to who?

25b) If you call a veterinarian, does he/she give you details about disease prevention, control, or protection?

25c) If you treat the animal yourself, how do you do that?

26) Do farmers share information about diseases or mass deaths in their livestock with other farmers?

26a) If yes, have other farmers encountered any disease outbreaks in their animals?

26b) If yes, which diseases have they encountered?

27) Do you think people in your community are aware of diseases they can catch from animals?

27a) If no, who do you think is responsible for improving awareness?

Options:

Community Health Workers

Veterinarians

Farmers

Government

Other

27b) If other, who is responsible?

28) Has anyone given you advice on how to minimize your risk of getting RVF?

28a) If yes, was it a man or woman?

28b) If yes, who gave you the advice?

Options:

Community Health Workers

Veterinarian

Government official

Farmer

28c) What did they tell you to do?

29) If you have received advice, did you implement it?

29a) If no, why did you not implement it?

30) Do you think women are credible sources of advice for farmers for issues concerning RVF risk mitigation or farming practices?

30a) If yes, what is the role of women in livestock diseases and RVF risk mitigation?

30b) What is the role of women in ensuring safe farming practices?

30c) If no, why aren't women credible sources of information about livestock diseases and RVF risk mitigation?

31) Do you think non-farmer community members (such as relatives or neighbors) are credible sources for advice on RVF and safe farming practices?

31a) If no, why not?

32) Are your animals vaccinated against RVF?

32a) If yes, which animals are vaccinated?

Options:

Goat

Cattle

Sheep

Chicken

Pig

Other

32b) If no, why aren't your animals vaccinated?

33) Have you ever been involved in any similar research, intervention, or government program concerned with RVF?

33a) If yes, when and what was it?

34) Are you okay with me recording your location?

Latitude and longitude

Submission Time
