# Supplementary material for: Rift Valley fever knowledge, mitigation strategies and communication preferences among male and female livestock farmers in Eastern Province, Rwanda
Source: PLoS Negl Trop Dis. 2021 Aug 23;15(8):e0009705. doi: 10.1371/journal.pntd.0009705 (PMC8412303; doi:10.1371/journal.pntd.0009705)

PLOS Neglected Tropical Diseases

A survey of knowledge, risk perceptions, mitigation strategies, and gender-nuanced communication practices among livestock farmers in Eastern Province following the first officially declared Rift Valley Fever outbreak in Rwanda

--Manuscript Draft--

**Manuscript Number:**	PNTD-D-21-00602
**Full Title:**	A survey of knowledge, risk perceptions, mitigation strategies, and gender-nuanced communication practices among livestock farmers in Eastern Province following the first officially declared Rift Valley Fever outbreak in Rwanda
**Short Title:**	Knowledge and communication among farmers in Rwanda following a Rift Valley Fever outbreak
**Article Type:**	Research Article
**Keywords:**	Rift Valley Fever; gender; Rwanda; knowledge; risk perceptions; communication practices; mitigation; information sharing; livestock farmers
**Abstract:**	The government of Rwanda reported an outbreak of Rift Valley Fever (RVF) in the Eastern Province in 2018. To respond to the outbreak, vaccination and education campaigns about the disease were carried out. Because RVF cases continue to be detected in Rwanda and the disease impacts livelihoods and health, accurate knowledge and communication are imperative. The objectives of this study were to evaluate knowledge and risk perceptions of RVF transmission among livestock farmers in Nyagatare District, Eastern Province, Rwanda, and to compare RVF knowledge, risk perceptions, and farming practices between male and female livestock farmers. This cross-sectional, quantitative study was conducted in four sectors of Nyagatare District in the Eastern Province of Rwanda in June 2019. A 34-question survey was used to ask about demographics, livestock ownership, risk perceptions about zoonotic diseases and livestock, RVF knowledge, preferred communication sources and information sharing strategies, and protective strategies for RVF mitigation while working with livestock. Livestock farmers were sampled exhaustively and interviewed at three milk collection centers, two village meeting points, a farm cooperative meeting, and during door-to-door visits in villages. In total, 123 livestock farmers were interviewed. The survey found that most livestock farmers lacked knowledge about epizootic and zoonotic transmission of RVF, more male livestock farmers were familiar with RVF and risk mitigation strategies, and female livestock farmers are not viewed as reliable sources of information. Additionally, most livestock farmers had not vaccinated their animals against RVF despite past vaccination campaigns and radio was the most popular communication channel. These findings show that RVF knowledge and information sharing are inadequate among livestock farmers in Eastern Province. Therefore, vaccination and education campaigns may need to be reevaluated within the context of these trends in order to increase coverage as well as information and vaccination uptake to prepare for future RVF outbreaks.
**Additional Information:**	
**Question**	**Response**
**Financial Disclosure**<br><br>Enter a financial disclosure statement that describes the sources of funding for the work included in this submission. Review the submission guidelines for detailed requirements. View published research articles from *PLOS NTDs* for specific examples.<br><br>This statement is required for submission	Authors and Funding: LS-Cummings Foundation, JS-University of Global Health Equity, AS-University of Global Health Equity, EN-Cummings Foundation, HA-Cummings Foundation and University of Global Health Equity.<br>Full names of Funders: Cummings Foundation and University of Global Health Equity.<br>Cummings Foundation Website URL: https://www.cummingsfoundation.org/<br>University of Global Health Equity Website URL: https://ughe.org/<br><br>NO. The funders had no role in study design, data collection and analysis, decision to publish, or preparation of the manuscript.

Powered by Editorial Manager® and ProduXion Manager® from Aries Systems Corporation

and **will appear in the published article** if the submission is accepted. Please make sure it is accurate.<br><br>**Unfunded studies**<br><br>Enter: *The author(s) received no specific funding for this work.*<br><br>**Funded studies**<br><br>Enter a statement with the following details:<br>• Initials of the authors who received each award<br>• Grant numbers awarded to each author<br>• The full name of each funder<br>• URL of each funder website<br>• Did the sponsors or funders play any role in the study design, data collection and analysis, decision to publish, or preparation of the manuscript?<br>• **NO** - Include this sentence at the end of your statement: *The funders had no role in study design, data collection and analysis, decision to publish, or preparation of the manuscript.*<br>• **YES** - Specify the role(s) played.<br><br>* typeset	
**Competing Interests**<br><br>Use the instructions below to enter a competing interest statement for this submission. On behalf of all authors, disclose any competing interests that could be perceived to bias this work—acknowledging all financial support and any other relevant financial or non-financial competing interests.<br><br>This statement **will appear in the published article** if the submission is accepted. Please make sure it is	The authors have declared that no competing interests exist.

accurate. View published research articles from *PLOS NTDs* for specific examples.<br><br>**NO authors have competing interests**<br><br>Enter: *The authors have declared that no competing interests exist.*<br><br>**Authors with competing interests**<br><br>Enter competing interest details beginning with this statement:<br><br>*I have read the journal's policy and the authors of this manuscript have the following competing interests: [insert competing interests here]*<br><br>* typeset<br><br>This statement is required for submission and **will appear in the published article** if the submission is accepted. Please make sure it is accurate and that any funding sources listed in your Funding Information later in the submission form are also declared in your Financial Disclosure statement.	
**Data Availability**<br><br>Authors are required to make all data underlying the findings described fully available, without restriction, and from the time of publication. PLOS allows rare	Yes - all data are fully available without restriction

exceptions to address legal and ethical concerns. See the PLOS Data Policy and FAQ for detailed information.<br><br>A Data Availability Statement describing where the data can be found is required at submission. Your answers to this question constitute the Data Availability Statement and **will be published in the article**, if accepted.<br><br>**Important:** Stating 'data available on request from the author' is not sufficient. If your data are only available upon request, select 'No' for the first question and explain your exceptional situation in the text box.<br><br>Do the authors confirm that all data underlying the findings described in their manuscript are fully available without restriction?	
**Describe where the data may be found in full sentences. If you are copying our sample text, replace any instances of XXX with the appropriate details.**<br><br>• If the data are **held or will be held in a public repository**, include URLs, accession numbers or DOIs. If this information will only be available after acceptance, indicate this by ticking the box below. For example: *All XXX files are available from the XXX database (accession number(s) XXX, XXX.)*.<br>• If the data are all contained **within the manuscript and/or Supporting Information files**, enter the following: *All relevant data are within the manuscript and its Supporting Information files.*<br>• If neither of these applies but you are	All relevant data are within the manuscript and its Supporting Information files.

able to provide **details of access elsewhere**, with or without limitations, please do so. For example:<br><br>*Data cannot be shared publicly because of [XXX]. Data are available from the XXX Institutional Data Access / Ethics Committee (contact via XXX) for researchers who meet the criteria for access to confidential data.*<br><br>*The data underlying the results presented in the study are available from (include the name of the third party and contact information or URL).*<br>• This text is appropriate if the data are owned by a third party and authors do not have permission to share the data.<br><br>* typeset	
Additional data availability information:	

Manuscript
Click here to access/download;Manuscript;Smith et al. RVF Knowledge Communication Rwanda Manuscript.docx

A survey of knowledge, risk perceptions, mitigation strategies, and gender-nuanced communication practices among livestock farmers in Eastern Province following the first officially declared Rift Valley Fever outbreak in Rwanda

Lindsay J. Smith[1*], Janna M. Schurer[1,2¶], Eurade Ntakiyisumba[3], Anselme Shyaka[3¶], J. Hellen Amuguni[1¶]

[1]Department of Infectious Disease and Global Health, Cummings School of Veterinary Medicine at Tufts University, North Grafton, MA, USA

[2]Center for One Health, University of Global Health Equity, Kigali, Rwanda

[3]School of Veterinary Medicine, College of Agriculture, Animal Sciences and Veterinary Medicine, University of Rwanda

*Corresponding Author

E-mail: Lindsay.Smith@tufts.edu

[¶]These authors co-supervised this study.

ABSTRACT

The government of Rwanda reported an outbreak of Rift Valley Fever (RVF) in the Eastern Province in 2018. To respond to the outbreak, vaccination and education campaigns about the disease were carried out. Because RVF cases continue to be detected in Rwanda and the disease impacts livelihoods and health, accurate knowledge and communication are imperative. The objectives of this study were to evaluate knowledge and risk perceptions of RVF transmission among livestock farmers in Nyagatare District, Eastern Province, Rwanda, and to compare RVF knowledge, risk perceptions, and farming practices between male and female livestock farmers. This cross-sectional, quantitative study was conducted in four sectors of Nyagatare District in the Eastern Province of Rwanda in June 2019. A 34-question survey was used to ask about demographics, livestock ownership, risk perceptions about zoonotic diseases and livestock, RVF knowledge, preferred communication sources and information sharing strategies, and protective strategies for RVF mitigation while working with livestock. Livestock farmers were sampled exhaustively and interviewed at three milk collection centers, two village meeting points, a farm cooperative meeting, and during door-to-door visits in villages. In total, 123 livestock farmers were interviewed. The survey found that most livestock farmers lacked knowledge about epizootic and zoonotic transmission of RVF, more male livestock farmers were familiar with RVF and risk mitigation strategies, and female livestock farmers are not viewed as reliable sources of information. Additionally, most livestock farmers had not vaccinated their animals against RVF despite past vaccination campaigns and radio was the most popular communication channel. These findings show that RVF knowledge and information sharing are inadequate among livestock farmers in Eastern Province. Therefore, vaccination and education campaigns

may need to be reevaluated within the context of these trends in order to increase coverage as well as information and vaccination uptake to prepare for future RVF outbreaks.

Keywords: Rift Valley Fever, gender, Rwanda, knowledge, risk perceptions, communication practices, mitigation, information sharing, livestock farmers

AUTHOR SUMMARY

This study was conducted in order to evaluate RVF knowledge and awareness as well as communication and mitigation strategies among livestock farmers in Eastern Province, Rwanda. Rwanda declared an outbreak of RVF in 2018 and cases have continued to be detected. Thus, evaluating the status of knowledge, preventive strategies, and information sharing among livestock farmers is crucial in mitigating future outbreaks. Our team conducted a survey of knowledge, risk perceptions, mitigation strategies, and communication practices among livestock farmers from four different sectors within Nyagatare District and compared them between male and female livestock farmers in order to analyze gender-nuanced differences between these groups. Sectors were chosen for sampling based on their proximity to previous outbreak areas. Survey questionnaire results showed that knowledge and risk perceptions differed between male and female livestock farmers, and that they could be generally improved among all livestock farmers. Female livestock farmers and non-farming community members were viewed as unreliable sources of information which could impact information dissemination. Many farmers also reported that their livestock herds were not vaccinated for the disease despite previous vaccination campaigns. Communication strategies and information sources also differed between

male and female livestock farmers, which highlights a need to consider gender in improving RVF vaccination and education campaign coverage. These findings pose implications for future community-based public health interventions as well as policy development for RVF control and mitigating future RVF outbreaks within Rwanda. The limitations of this study were the small sample size and small geographic area, as well as potential biases in survey responses.

INTRODUCTION

Rift Valley Fever (RVF) is a mosquito-borne viral zoonotic disease caused by a phlebovirus of the family *Phenuiviridae* [1,2]. RVF is spread among mosquitos by vertical transmission [3,4]. Ruminants, such as buffalo, cattle, sheep, and goats, become infected via mosquito bites, direct contact with infected ruminants, and indirect contact via contaminated fomites [1,2]. Many RVF outbreaks have occurred throughout eastern Africa [5]. They have had notable impacts in countries such as Kenya and Uganda, which have experienced massive livestock losses and numerous human cases of the disease [5]. Clinical signs in animals include newborn mortality, mass abortions, sudden decreased milk production, excessive regurgitation, high fever, diarrhea, bleeding from body orifices, anorexia and lethargy, and, sometimes, death [6,7]. Clinical signs and symptoms in humans include high fever, headache, back pain, loss of vision, and bleeding from body orifices [6,7]. This spectrum of symptoms range in both people and animals from being asymptomatic to hemorrhagic fever and death [7]. Risk factors associated with zoonotic transmission of RVF include working closely with ruminants, birthing animals, skinning and slaughtering animals, drinking raw milk, sheltering animals in the home, not wearing protective equipment or disinfecting after handling infected animals, and milking infected animals [8—10].

The animal, human, and environmental drivers of RVF make mitigating outbreaks of this disease a One Health challenge [4,11—13].

Rwanda, a country with extensive cattle production, reported an RVF outbreak in Eastern Province in 2018 following the 2017 outbreak in Niger and the widespread 2007 outbreaks in Sudan, Kenya, Somalia, and Tanzania [14]. Additional RVF cases detected in Rwanda, with many in Eastern Province, throughout 2020 signify a continuing challenge. According to the OIE World Animal Health Information System, sporadic clusters of RVF cases had been detected in Rwanda and the virus had likely been circulating since 2012 [15]. Although serology has detected RVF cases in Rwanda since 2012, an RVF outbreak was not officially reported by authorities in the country until 2018 [15]. The 2018 outbreak was the first RVF outbreak to be officially recognized and declared by the government of Rwanda.

In 2018, livestock cases were reported in eight sectors across five districts (Ngoma, Kirehe, Rwamagana, Kayonza, Gatsibo) [16,17]. The outbreak started in Ngoma and Kirehe at the end of May 2018, and many cases continued to be reported through the end of July 2018 [18]. The outbreak spread to these other districts, so animal movements were banned from mid-June to the end of July to mitigate the outbreak. Two veterinary officers died from the disease, but widespread human cases were not reported [18]. Hundreds of cattle aborted throughout this outbreak, and cases were confirmed via enzyme-linked immunosorbent assay (ELISA) in the national veterinary laboratory of Rubirizi [14, 16, 17]. The Rwanda Agriculture Board responded by vaccinating 237,386 cattle, 22,727 goats, and 17,872 sheep against RVF [16]. The public sectors implemented vector control, ante- and post-mortem inspections, and public awareness

campaigns [16, 17]. These interventions helped to contain the outbreak, mitigate further losses of cattle, and allow time to improve national laboratory capacities for case detection [16,17]. The vaccination campaign of healthy and nonpregnant animals interrupted transmission and ended the outbreak in July 2018 [18].

Throughout 2020, Rwanda experienced 32 outbreaks of RVF across the country with 689 total cases recorded in ruminant livestock [19]. Nyagatare experienced three outbreaks in January and one in March, with 213 total cases [19]. In other parts of Eastern Province, specifically Gatsibo and Ngoma Districts, a total of 109 RVF cases were recorded in ruminant livestock species [19]. In response to these RVF outbreaks in 2020, a total of 354,380 animals were documented as being vaccinated for RVF around outbreak areas, and Eastern and Southern Province as well as districts around Kigali were most heavily affected [19].

Cattle have great cultural and economic significance in Rwanda while sheep and goats are widespread and have potential to advance livestock sector development. Traditionally, marriage in Rwanda is marked by the presentation of a bride dowry in the form of cattle. The Girinka Program, or the One Cow per Family Program, provides a free cow to a poor family to reduce childhood malnutrition and raise rural incomes through milk sales [20]. Additionally, 11% of Rwanda’s GDP came from livestock and livestock products in the fourth quarter of 2019 [21,22]. Small ruminants have been reported to suffer higher mortality from RVF than cattle, but sheep and goat meat production are to increase by 33% and 50% by 2021/22, respectively [23, 24]. While cattle is projected to supply 72% of red meat in Rwanda for 2021/22, economic analyses have identified red meat and milk from cattle, sheep, and goats as having the most potential for

productivity increases through new investment, and for contributing most to long-term development of the livestock sector and value chains [23]. In 2017 it was estimated that there were more than twice as many goats in Rwanda as cattle [23]. Because RVF causes high mortality rates among young ruminants, mass abortions in older animals, and high morbidity rates, it is a major threat to the livestock production sector and to the economic wellbeing of livestock farmers.

Livestock farmer behaviors and risk perceptions play a key role in controlling zoonotic diseases. During RVF outbreaks in South Africa, Mayotte, Tanzania, West Africa, and Kenya, most human RVF cases were attributed to livestock farmers handling or consuming infected livestock products rather than by mosquito bites [25]. Livestock farmers also spread it between animals via contaminated fomites such as calving and artificial insemination equipment [1,2,26]. Thus, in order to mitigate future RVF outbreaks in Rwanda, the behavioral risk perceptions of livestock farmers must be understood. This information is important because it influences participation in livestock vaccination campaigns, vector control, disease reporting, and risk avoidant behaviors, while informing educational and preventive measures to maintain productivity of livestock and encourage safer husbandry practices. Livestock trade is highlighted as a path to introduce RVF into non-endemic areas as well, with illegal cross-border cattle movement being a concern [27]. Infected cattle that produce less milk and cannot be moved or traded hinder poor families instead of raising incomes, with trade and consumption bans posing dietary and livelihood challenges to poor communities [25]. To address these challenges, knowledge gaps and behavior patterns need to be identified to inform and design effective disease mitigation and communication campaigns. Studies show that livestock farmers and butchers in East African countries are more likely to

recognize that they are at risk for contracting RVF, but that many do not know the full range of RVF symptoms to look for in humans or animals, nor wear personal protective equipment [27—30]. Additionally, male and female livestock farmers have different farming experiences, and understanding gender differences in knowledge and risk perceptions can maximize intervention efficacy across audiences. Interventions that engage and change risk perceptions increase health behaviors, which can prevent future RVF outbreaks and mitigate transmission [29, 30].

As a result of climate change and shifting mosquito ranges, RVF is spreading to new ecologies and environments while threatening rural livelihoods and health [31]. Using behavioral risk perceptions of RVF among high risk populations like livestock farmers can provide local and national governments with entry points and information about what to target within effective educational campaigns. There are no studies of livestock farmer knowledge or risk perceptions regarding RVF in Rwanda. Thus, the specific aims of this study were to (1) evaluate knowledge and risk perceptions of RVF transmission among livestock farmers in Nyagatare District, Eastern Province, Rwanda, and (2) to compare RVF knowledge, risk perceptions, and farming practices between male and female livestock farmers.

METHODS

Description of the Study Area

This study was conducted in four sectors of Nyagatare District in the Eastern Province of Rwanda, with most participants living in Rwimiyaga and Rwempasha, and others living in Nyagatare and Rukomo sectors (Figure 1). These sectors were chosen due to their proximity to sectors with RVF outbreaks. Rwimiyaga reported cases of RVF in 2018, while Rwempasha did not. Nyagatare and Rukomo were unaffected but are located close to outbreak areas [16,17]. The

estimated total human population of Nyagatare District, using data from 2011 Integrated Household Living Conditions Survey (EICV3) and national population medium-level growth projections between 2012 and 2020, is approximated to be 512,200 [32]. Nyagatare District is comprised of 14 sectors in total. Using sector population estimates from 2012 and the medium-level projected national population growth for Rwanda between 2012 and 2020, it can be estimated that the population of Rwimiyaga is now approximately 69,000, Rwempasha is 24,600, Nyagatare is 62,500, and Rukomo is 41,000 [33]. The largest source of employment in Nyagatare is agriculture with 79.6% of men and women aged 16 years or older engaged in cash crop or livestock production (primarily cattle, goats, sheep, chickens) [33]. Most men and women in Nyagatare are small-scale farm owners (57% and 74.5%, respectively) or hourly waged farm workers (13% each) [23]. Cattle production systems are extensive or semi-intensive with low levels of intensification [23]. Nyagatare District and its corresponding cattle grazing areas are located adjacent to extensive stagnant water in rice wetlands, which can serve as mosquito breeding sites [1,2,12,16].

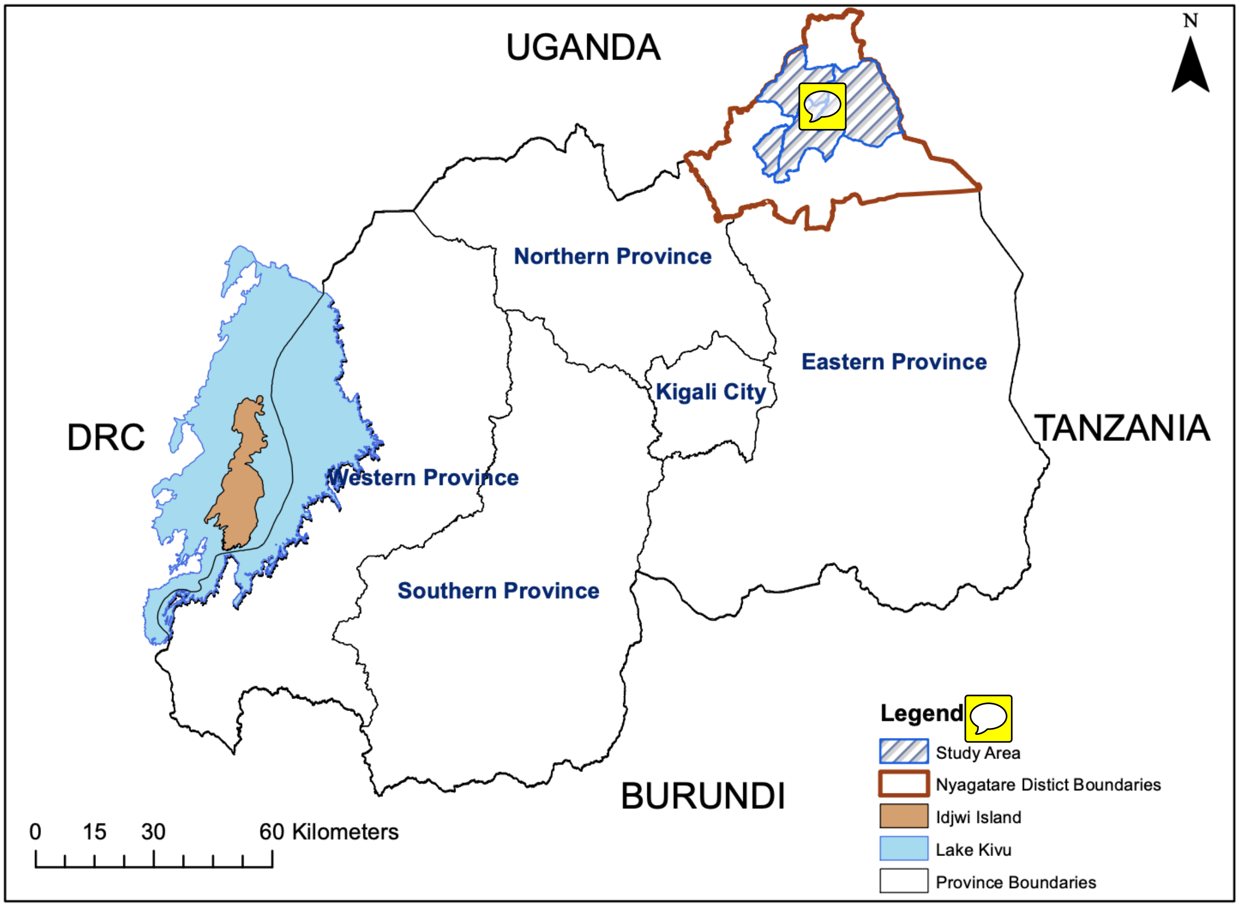

Figure 1. Map of Study Area

Study Design and Data Collection

A cross-sectional, quantitative study was conducted in June 2019. The research team sampled exhaustively by approaching prospective participants at three milk collection centers in Rwimiyaga, Rwempasha and Nyagatare, two village meeting points in Rwempasha, a farm cooperative meeting in Rwimiyaga, and during door-to-door visits in villages in Rwempasha until no additional participants could be found (Table 1). In total, 123 livestock farmers from four sectors of Nyagatare District were interviewed. The inclusion criteria was ruminant livestock ownership and being an adult that was 18 years of age or older.

Table 1. Number of Participants Interviewed at Each Meeting Location

Meeting location	Administrative locality (area)	Number of participants
Milk collection center	Rwimiyaga, Rwempasha, Nyagatare	62
Village meeting point	Rwempasha	19
Cooperative meeting	Rwimiyaga	22
Door-to-door	Rwempasha	20

The questionnaire included 34 questions and six parts: a) demographics, b) livestock ownership, c) risk perceptions about zoonotic diseases and livestock, d) RVF knowledge e) preferred communication sources and information sharing strategies, and f) protective strategies for RVF mitigation while working with livestock. It was written in English and then translated to Kinyarwanda, with interviews conducted in Kinyarwanda. A mixture of yes/no, open-ended, and option selection questions were used. Two "yes" or "no" questions focusing on specific RVF transmission knowledge were only asked to respondents who had already heard of RVF. Follow-up questions were asked to participants to verify their knowledge of transmission routes.

The questionnaire was pre-tested on a group of volunteers from Nyagatare town, adjusted as needed before its upload onto mobile phones using the Kobo Toolbox application (https://www.kobotoolbox.org/). Livestock farmer responses were directly entered into Kobo Toolbox in English, uploaded to the server and checked for errors daily.

Statistical Analysis

Frequencies for responses were calculated using SPSSv26 (IBM, United States) statistical software. Reponses to all questions were reported descriptively. Fisher's Exact Test was used to test for association between respondent gender or administrative locality and respondent knowledge or communication preferences. The cutoff for statistical significance was 5%. In

order to establish age group for participant demographics, total age range was divided into quintiles.

Ethics Statement

Verbal consent was obtained from participants prior to every interview. The data were analyzed anonymously. Ethical approval for this study was granted by the Institutional Review Board at Tufts University, Massachusetts, USA, and by the Research Screening and Ethical Clearance Committee of the College of Agriculture, Animal Sciences, and Veterinary Medicine at the University of Rwanda (Ethical approval reference: 025/17/DRIPGS). Through the Office of the Vice Provost for Research at Tufts University, this study was granted the status of Exempt Category 2 as defined in 45 CFR 46.104 (d). Details for this can be found on the Office for Human Research Protections (OHRP) website at: http://www.hhs.gov/ohrp/humansubjects/guidance/45cfr46.html#46.104(d). This study was given the identification of IRB Study #1955018 with the status of Exempt through the Social, Behavioral, and Educational Research Institutional Review Board within the Office of the Vice Provost for Research at Tufts University, Massachusetts, USA.

RESULTS

Participant Demographics

In this study, 62 (50.4%) women and 61 (49.6%) men were interviewed, with most participants aged between 22 and 53 years (35.8% between 22 and 37, 38.2% between 38 and 53; Table 2). Participants most often owned cattle and/or goats (92.7%) versus other animals such as chickens,

pigs, sheep, dogs, cats, and rabbits. Three-quarters of participants (75.6%) had no education or had completed primary school; two-thirds (66.7%) had raised livestock for over 10 years.

Table 2. Demographic profile of Respondents from Nyagatare District, Rwanda (N=123)

Variable	Frequency n (%)
**Gender**	
Female	62 (50.4)
Male	61 (49.6)
**Age quintiles (years)**	
22—37	44 (35.8)
38—53	47 (38.2)
54—69	28 (22.8)
70—85	2 (1.6)
>85	2 (1.6)
**Education Level**	
No formal education	38 (30.9)
Primary	55 (44.7)
Secondary	26 (21.1)
University	4 (3.3)
**Sector**	
Nyagatare	13 (10.6)
Rukomo	1 (0.8)
Rwempasha	45 (36.6)
Rwimiyaga	64 (52.0)
**Years Raising Livestock**	
<1	8 (6.5)
1-5	24 (19.5)
6-10	9 (7.3)
> 10	82 (66.7)
**Animals Owned**[1]	
Cattle	114 (92.7)
Goat	64 (52.0)
Chicken	22 (17.9)
Sheep	14 (11.4)
Pig	3 (2.4)
Other[2]	3 (2.4)

[1] Respondents could own multiple animal types;[2]Other- dogs, cats, rabbits

Knowledge and Risk Perceptions of Rift Valley Fever

Overall, more male participants (78.7%) had heard of RVF than female participants (59.7%, p=0.031) and more men than women had received advice on minimizing the risk of infection (37.7% versus 12.9%; p=0.002; Table 3). Less than half were aware that people could be infected (43.0%) and few understood how livestock were infected (15.4%). Upon answering follow-up questions, approximately half of participants (43.9%) could name specific zoonotic diseases (Table 3). Most frequently named zoonotic diseases were foot and mouth disease, tuberculosis, brucellosis, anthrax, and intestinal parasites, and of the participants who named zoonotic diseases, only 5% named RVF. Educational level was statistically associated with perception of risk associated with livestock rearing (p=0.020).

Table 3: Knowledge and Risk Perceptions of Rift Valley Fever Among Participants from Nyagatare District, Rwanda (N=123)

	Male	Female	Total	Fisher Test p-value
	n (%)			
Has heard of RVF				
Yes	48 (78.7)	37 (59.7)	85 (69.1)	
No	13 (21.3)	25 (40.3)	38 (30.9)	0.031
Knows humans can be infected with RVF (n=85)				
Yes	20 (41.7)	17 (45.9)	37 (43.5)	
No	28 (58.3)	20 (54.1)	48 (56.5)	0.778
Knows how livestock become infected with RVF (n=85)				
Yes	11 (22.9)	8 (21.6)	19 (22.4)	
No	37 (77.1)	29 (78.4)	66 (77.6)	0.927
Has received advice on minimizing risk of RVF				
Yes	23 (37.7)	8 (12.9)	31 (25.2)	
No	38 (62.3)	54 (87.1)	92 (74.8)	0.002
Believes tending livestock poses health risks				
Yes	30 (49.2)	25 (40.3)	55 (44.7)	0.367
No	31 (50.8)	37 (59.7)	68 (55.3)	
Is aware of specific zoonotic diseases transmissible from livestock to people				

Yes<br>No	35 (57.4)<br>26 (42.6)	32 (51.6)<br>30 (48.4)	67 (54.5)<br>56 (45.5)	0.588

Risk Mitigation Efforts for Rift Valley Fever

Approximately half of participants (44.7%) believed that tending livestock posed health risks and 25.2% took measures to protect themselves while working with animals (Table 4). More men slaughtered their livestock than women (32.8% versus 12.9%, p=0.010) and the majority (92.9%) of respondents who slaughtered their own animals reported not wearing personal protective equipment when slaughtering animals. Only one in four participants (22%) had vaccinated livestock against RVF (Table 4). Educational level was not associated with using protective measures (p=0.267)

Table 4. Risk Mitigation Efforts for Rift Valley Fever Among Participants from Nyagatare District, Rwanda (N=123)

	Male	Female	Total	Fisher Test p-value
	n (%)			
Wears personal protective equipment when slaughtering animals				
Yes<br>No	2 (10.0)<br>18 (90.0)	0<br>8 (100.0)	2 (7.1)<br>26 (92.9)	1.00
Slaughters his/her own animals				
Yes<br>No	20 (32.8)<br>41 (67.2)	8 (12.9)<br>54 (87.1)	28 (22.8)<br>95 (77.2)	0.010
Takes protective measures while working with animals				
Yes<br>No	18 (29.5)<br>43 (70.5)	13 (21.0)<br>49 (79.0)	31 (25.2)<br>92 (74.8)	0.305
Animals are vaccinated against RVF				
Yes<br>No	18 (29.5)<br>43 (70.5)	9 (14.5)<br>53 (85.5)	27 (22.0)<br>96 (78.0)	0.052

Communication Practices and Preferences

Most participants (88.6%) heard about disease outbreaks on the radio (Table 5). Respondents most often preferred obtaining information on the radio (33%), followed by the telephone (27.3%), face-to-face (23.4%), newspapers (1.9%) and other (14.4%), which included community meetings, veterinarians, cooperatives, and community health workers. There were no significant differences between men and women with respect to communication preferences.

Table 5. Preferred Sources of Information among Respondents in Nyagatare District, Rwanda (N=123)

	Male	Female	Total	Fisher Test p-value
	n (%)			
Hears about disease outbreaks on the radio				
Yes	55 (90.1)	54 (87.1)	109 (88.6)	
No	6 (9.9)	8 (12.9)	14 (11.4)	0.778
What is your preferred method(s) of receiving information?[3]				
Radio	32 (29.4)	37 (37.0)	69 (33.0)	0.129
Telephone	30 (27.5)	27 (27.0)	57 (27.3)	
Talking in person	30 (27.5)	19 (19.0)	49 (23.4)	
Newspapers	1 (0.9)	3 (3.0)	4 (1.9)	
Other[4]	16 (14.7)	14 (14.0)	30 (14.4)	

[3]Respondents could select more than one preferred method of receiving information.

[4]Other forms of communication included community and farmer meetings, television, livestock farmers, veterinarians, community health workers, someone with training on farming techniques, cooperatives.

Attitudes toward Source of Information

More women (83.9%) than men (63.9%) felt women were credible sources of advice about crop production and raising livestock (p=0.014). Approximately half (52%) of respondents felt that non-livestock farmers could provide credible crop production- and livestock-related information (Table 6). There was no significant difference between the attitudes of male and female livestock farmers toward the credibility of information from non-livestock farmers.

Table 6. Attitudes toward Source of Information among Respondents in Nyagatare District, Rwanda (N=123)

	Male	Female	Total	Fisher Test p-value
	n (%)			
Thinks women are credible sources of advice				
Yes<br>No	39 (63.9)<br>22 (36.1)	52 (83.9)<br>10 (16.1)	91 (74.0)<br>32 (26.0)	0.014
Thinks non-livestock farmers can be credible sources of information				
Yes<br>No	29 (47.5)<br>32 (52.5)	35 (56.5)<br>27 (43.5)	64 (52.0)<br>59 (48.0)	0.369

Rift Valley Fever Knowledge across Rwimiyaga and Rwempasha Sectors

Participants differed between sectors in zoonosis awareness (p=0.007), with 36.7% of respondents from Rwimiyaga saying livestock can transmit disease, versus 14.7% of respondents from Rwempasha. When faced with ill livestock, Rwimiyaga participants were more likely to call a veterinarian than Rwempasha participants (43.1% and 23.9% respectively, p=0.030). There was no significant difference between sectors regarding whether participants had heard of RVF, knew if people can be infected with RVF, or knew how livestock become infected with the disease. Sector was also not significantly related to wearing personal protective equipment when slaughtering animals or having ever had animals vaccinated for RVF.

DISCUSSION

Our study of livestock farmers living adjacent to RVF outbreak areas highlighted generally poor knowledge of RVF and its zoonotic nature among livestock farmers, poor vaccination penetration, significant differences between men and women in RVF knowledge and risk mitigation as well as preferred sources of livestock and disease prevention advice, and radio being the most preferred communication channel. The differences found between male and female livestock farmers highlight the gendered dimensions of education campaigns, access to information, and knowledge uptake. Because RVF outbreaks continue to occur in Eastern Province and throughout Rwanda, these trends present important considerations for behavior change, zoonotic transmission prevention, and outbreak mitigation interventions.

Because many livestock farmers reported being familiar with RVF but did not have accurate knowledge about epizootic or zoonotic transmission, livestock farmers may be unaware of their lack of knowledge, and because of this they may not seek nor be receptive to more accurate, up-to-date-information [34,35]. Additionally, low levels of awareness and knowledge have been identified results of communication inequality and accessibility, which alter exposure to and success of public health messaging [34, 36]. Thus, accessibility, frequency, and distribution of education campaigns could create barriers to information dissemination in communities, especially in rural areas and villages in Eastern Province. Experience with active RVF outbreaks could also influence retention of accurate, thorough RVF knowledge, and the relatively smaller number of outbreaks in Rwanda compared to other countries could reduce awareness and knowledge. A study on knowledge, attitudes, and practices among farmers and slaughterhouse workers in Uganda found that while many farmers and butchers recognized RVF, less than half of participants could accurately describe transmission between animals and from animals to humans [36]. Uganda has experienced fewer RVF outbreaks than Kenya, where larger and more

frequent RVF outbreaks have occurred with significant economic impacts and people there have demonstrated more thorough knowledge of the disease [37,38]. In the Eastern Province of Rwanda, RVF knowledge may be weak despite previous vaccination and education campaigns because many livestock farmers may have not yet experienced RVF in their animals or its economic impacts directly [38]. This may decrease the level of RVF knowledge that farmers have and apply over time. Furthermore, covering adequate areas of Eastern Province including rural villages while also spending adequate time thoroughly educating livestock farmers can be challenging and lead to either coverage gaps or insufficient knowledge.

Despite past vaccination campaigns in Rwanda, many livestock farmers surveyed in this study reported that their animals were not vaccinated for RVF. Vaccination campaigns may not be sufficiently covering the Eastern Province, which could be due to financing, supply chain, and policy challenges [39]. Numerous service delivery challenges could exist and present barriers to penetration of the vaccine across rural areas [39]. Inadequate infrastructure for distribution, cold chain, and transport could lead to decreased vaccine coverage [39]. Stockpiling of vaccines and limited supply can affect coverage as well, and logistics of vaccination campaign rollout, such as locations targeted in vaccination campaigns as well as timing and methods of information sharing, can limit the amount of farmers who are able to take their livestock to get vaccinated [39, 40]. The high number of livestock farmers reporting that their livestock are not vaccinated shows that these issues may be limiting the capacity of vaccine distribution [39]. Continued outbreaks of RVF could place more strain on the supply of vaccines and lead to limited coverage during vaccination campaigns.

Differences between men and women in RVF knowledge, understanding of RVF zoonotic transmission, and receiving advice on RVF risk mitigation show different access to information.

Additionally, different preferences on who to seek advice from highlight gendered perceptions of reliability of other livestock farmers. More male livestock farmers had heard of RVF and received advice on mitigating zoonotic transmission of the disease than female livestock farmers. In Rwanda, men manage cows and cash crops while women manage small animals such as goats and chickens [41]. Additionally, RVF education campaigns in Rwanda are done prior to when livestock, mostly cattle, get vaccinated. Cattle keepers and owners, the vast majority of whom are men, the ones bringing cows for vaccination and thus they are more likely to benefit from the RVF education campaigns and know more about the disease. Furthermore, 79.1% of women are involved in agriculture, with most being subsistence agriculture, and few women belong to agricultural cooperatives or hold leadership roles [42]. Women's household labor responsibilities and their limited livestock ownership rights, decision-making power, and control over income make them less likely to work with cattle and therefore less likely to gain knowledge and vaccinations from RVF campaigns [43—46]. Additionally, more male farmers reported slaughtering their own animals, thus they may be more knowledgeable of RVF since they are more directly at risk with this activity [35,37]. This structure of intra-household decision-making and income control presents barriers to vaccination and information uptake at the community level, which has implications for long-term RVF control in Rwanda [44—49]. In past educational campaigns, RVF information was informally presented before, during, and after vaccination campaigns, with local veterinarians reporting numbers of cattle vaccinated and money paid to vaccination assistants. No further documentation is produced. If women miss educational campaigns, participants and species vaccinated are not documented, and women do not discuss RVF with male farmers, knowledge and vaccination gaps may complicate RVF control in Rwanda.

Radio was most preferred for disease outbreak communications, which could be due to its widespread accessibility via networks throughout Rwanda. According to the Fifth Integrated Household Living Conditions Survey by the National Institute of Statistics of Rwanda, 73.1 % of households in Eastern Province own a radio and 66.1% own a mobile phone [50]. A study in Rwanda conducted by the Consultative Group on International Agricultural Research reported that male spouses typically owned radios in Eastern Province [51]. Previous studies show that educational campaigns on radio and mobile phones rapidly disseminate information and change behavior, making campaigns crucial interventions [51,52]. Intervening in an outbreak early with educational campaigns has shown to decrease disease spread during outbreaks [52]. Additionally, a previous study on disseminating nutritional information repeatedly over radio found that this changed dietary behaviors in Rwanda [54]. Another found that men and women were highly engaged in a radio drama aimed at improving family planning and that it encouraged conversation [55]. Because radio has been used for past educational campaigns and communities have heard disease outbreak information on the radio before, radio is effective and livestock farmers prefer it as a reliable channel through which to receive information.

Finally, the previous RVF outbreak in Rwimiyaga may have increased awareness of zoonotic diseases and tendency to call a veterinarian among livestock farmers there because of their direct experience with the disease [36,37]. More livestock farmers in Rwimiyaga may have had experience with previous RVF vaccination and education campaigns which increased their general awareness of zoonotic diseases. Because Rwempasha had not experienced an RVF outbreak, livestock farmers there may not have received RVF education nor general information on zoonotic disease risks. Educational campaigns may have been much less extensive in

Rwempasha with less information sharing among livestock there. Additionally, education levels could have been different among livestock farmers in Rwimiyaga and Rwempasha, leading to different levels of understanding of zoonotic transmission and the importance of contacting veterinarians when animals are sick. Herd sizes could lead to different levels of zoonosis knowledge among livestock farmers in Rwimiyaga and Rwempasha [28,56]. Participants from Rwimiyaga may have owned larger livestock herds, therefore having more experience with disease management and more motivation to pursue education and vaccination, as was found in studies about RVF and cattle trypanosomiasis Kenya and West Africa [28, 56].

These findings pose several implications for RVF awareness and control in the Eastern Province of Rwanda. Knowledge about RVF and its transmission needs to improve so that livestock farmers can protect themselves and their animals from outbreaks and pursue vaccination. Additionally, improving logistics and information dissemination about vaccination campaigns can increase vaccination penetration. Gendered differences in livestock ownership and information access should be evaluated to improve RVF knowledge and vaccination. For example, female livestock farmers tend to own small ruminants like goats and sheep instead of cattle [41,43-46]. With cattle and male cattle owners being primarily targeted during campaigns, small ruminants will remain susceptible to RVF and also spread the virus to cattle. Female livestock farmers' lack of knowledge can expose them to RVF as a zoonosis and enhance transmission of RVF between small ruminants and cattle, especially as small ruminants are less likely to be vaccinated. Livestock farmers with larger herds may be more likely to pursue RVF education and vaccination, and smaller herds as well as goats and sheep may be missed in campaigns and continue to circulate the virus [24, 27, 56]. Finally, because radio is so popular,

information spread on other communication channels may not be as successful in reaching livestock farmers, or the current frequency of information dissemination via radio may need to increase in order to improve knowledge retention among livestock farmers. All of these factors should be considered in designing future RVF education and vaccination campaigns to increase coverage and improve knowledge, especially since RVF outbreaks continue to occur in Eastern Province.

There were several limitations to this study. First, our study population was relatively small. Analyses were done with a Fisher's Exact Test to accommodate the small sample size. This study compared respondents in a small geographic area in Rwanda. Lastly, because this was a survey-based study asking respondents about their knowledge and practices to prevent RVF transmission, there could be bias in answers due to respondents wanting to appear responsible.

Conclusions and Recommendations

Our team has several evidence-based recommendations for developing future interventions for RVF in Rwanda. Gendered approaches to radio-based information dissemination should be considered, as radio has found to be preferred and effective [35, 38]. RVF educational campaigns should be evaluated on the ground to ensure that information is disseminated to both women and men. Further documentation of campaign participant demographics and information disseminated should be required to ensure adequate coverage. Targeted interventions maximize potential for behavior change, which is instrumental in mitigating RVF outbreaks.

RVF control is effective when integrated measures are deployed, relying on case management, surveillance, community education, preventive measures, and laboratory diagnosis [57]. RVF control contributes to Rwanda's policy goals of improving food security and family incomes by protecting cattle herd health and productivity. Because Rwanda experienced its first officially reported RVF outbreak in 2018 and the disease continues to re-emerge, this information can shape interventions. Educational campaigns can encourage vaccination, vector control, and personal protection, and serve as the first step in mitigating zoonotic disease impacts within Eastern Province. On a global scale, this will advance the Sustainable Development Goals of reducing poverty, eradicating hunger, promoting good health, and improving gender equality.

ACKNOWLEDGEMENTS

The authors wish to acknowledge Nyagatare district and sector veterinary officers, community leaders and members, community health workers, and Radio Nyagatare. They sincerely thank Hyacinthe Dusingize for her significant contributions to survey questionnaire development and data collection, as well as for conducting surveys. Finally, they extend gratitude to the Cummings School of Veterinary Medicine, the University of Rwanda College of Agriculture, Animal Sciences, and Veterinary Medicine, and the Tufts-UGHE-UR One Health Collaborative for supporting this project and aiding in its publication. This work was supported by grants from Cummings Foundation and the University of Global Health Equity, Rwanda.

REFERENCES

1. Javelle E, Lesueur A, Pommier de Santi V, de Laval F, Lefebvre T, Holweck G, et al. The challenging management of Rift Valley Fever in humans: literature review of the clinical disease and algorithm proposal. Ann Clin Microbiol Antimicrob [Internet]. 2020 Jan 22 [cited 2020 Nov 14];19. Available from: https://www.ncbi.nlm.nih.gov/pmc/articles/PMC6977312/
2. Lumley S, Horton DL, Hernandez-Triana LLM, Johnson N, Fooks AR, Hewson R. Rift Valley fever virus: strategies for maintenance, survival and vertical transmission in mosquitoes. J Gen Virol. 2017 May;98(5):875–87.
3. Glancey MM, Anyamba A, Linthicum KJ. Epidemiologic and Environmental Risk Factors of Rift Valley Fever in Southern Africa from 2008 to 2011. Vector-Borne and Zoonotic Diseases. 2015 Aug;15(8):502–11.
4. Lessons from the 2006–2007 Rift Valley fever outbreak in East Africa: implications for prevention of emerging infectious diseases. Future Virology. [cited 2020 Sep 7]. Available from: https://www.futuremedicine.com/doi/full/10.2217/17460794.3.5.411
5. CDC. Outbreak Summaries, Rift Valley Fever. [cited 2020 Jun 1]. Available from: https://www.cdc.gov/vhf/rvf/outbreaks/summaries.html
6. Laughlin LW, Meegan JM, Strausbaugh LJ, Morens DM, Watten RH. Epidemic Rift Valley fever in Egypt: observations of the spectrum of human illness. Trans R Soc Trop Med Hyg. 1979;73(6):630–3.
7. Wilson ML. Rift Valley fever virus ecology and the epidemiology of disease emergence. Ann N Y Acad Sci. 1994 Dec 15;740:169–80.

8. Mohamed M, Mosha F, Mghamba J, Zaki SR, Shieh W-J, Paweska J, et al. Epidemiologic and Clinical Aspects of a Rift Valley Fever Outbreak in Humans in Tanzania, 2007. Am J Trop Med Hyg. 2010 Aug 5;83(2 Suppl):22–7.
9. CDC. Rift Valley Fever | Disease Directory | Travelers' Health. [cited 2020 Apr 11]. Available from: https://wwwnc.cdc.gov/travel/diseases/rift-river-valley
10. Nicholas DE, Jacobsen KH, Waters NM. Risk factors associated with human Rift Valley fever infection: systematic review and meta-analysis. Tropical Medicine & International Health. 2014;19(12):1420–9.
11. The Historical and Recent Impact of Rift Valley Fever in Africa. [cited 2020 Sep 7]. Available from: https://www.ncbi.nlm.nih.gov/pmc/articles/PMC2913498/
12. Umuhoza T, Berkvens D, Gafarasi I, Rukelibuga J, Mushonga B, Biryomumaisho S. Seroprevalence of Rift Valley fever in cattle along the Akagera-Nyabarongo rivers, Rwanda. Journal of the South African Veterinary Association. 2017;88(1):1–5.
13. Nanyingi MO, Munyua P, Kiama SG, Muchemi GM, Thumbi SM, Bitek AO, et al. A systematic review of Rift Valley Fever epidemiology 1931–2014. Infect Ecol Epidemiol [Internet]. 2015 Jul 31 [cited 2020 May 3];5. Available from: https://www.ncbi.nlm.nih.gov/pmc/articles/PMC4522434/
14. Overseas Advisory Council. Health Alert: Kigali (Rwanda), Rift Valley Fever Outbreak in East Province [Internet]. Washington, D.C.: U.S. Department of State Bureau of Diplomatic Security; 2018 Jun. Available from: https://www.osac.gov/Content/Report/a28b4829-7a91-4908-9c85-15f4ae72fd19

15. OIE World Animal Health Information System. World Organization for Animal Health Disease Notifications [Internet]. Rwanda; 2021 [cited 2021 Apr 8]. Available from: https://wahis.oie.int/#/home

16. OIE. Rift Valley Fever, Rwanda, Information received on 10/08/2018 [Internet]. [cited 2020 Apr 12]. Available from: https://www.oie.int/wahis_2/public/wahid.php/Reviewreport/Review?page_refer=MapFullEventReport&reportid=27540

17. Rwanda Declares Quarantine as Deadly Rift Valley Fever Claims 99 Cattle – KT PRESS [Internet]. [cited 2020 Apr 12]. Available from: https://www.ktpress.rw/2018/06/rwanda-declares-quarantine-as-deadly-rift-valley-fever-claims-99-cattle/

18. Identification of Bunyamwera and Possible Other Orthobunyavirus Infections and Disease in Cattle during a Rift Valley Fever Outbreak in Rwanda in 2018 | The American Journal of Tropical Medicine and Hygiene [Internet]. [cited 2020 Nov 15]. Available from: https://www.ajtmh.org/content/journals/10.4269/ajtmh.19-0596

19. Rwanda Agriculture and Animal Resources Development Board. Rwanda Animal Health Report [Internet]. Kigali, Rwanda: Rwanda Agriculture Board; 2020; pp 1–32.

20. Umuzigambeho JF. Gender Assessment of Dairy Value Chains: Evidence from Rwanda. Rome, Italy: Food and Agriculture Organization of the United Nations; 2017.

21. GDP National Accounts (First Quarter 2020) | National Institute of Statistics Rwanda [Internet]. [cited 2020 Sep 17]. Available from: https://www.statistics.gov.rw/publication/gdp-national-accounts-first-quarter-2020

22. World Bank. International Development Association Program Appraisal Document on a Proposed Credit in the Amount of SDR 65.9 Million to the Republic of Rwanda [Internet].

The World Bank; 2015 [cited 2020 Apr 12]. Available from: http://elibrary.worldbank.org/doi/book/10.1596/978-1-4648-0484-7

23. Shapiro B, Getachew G, Desta S, Nigussie K. Rwanda Livestock Master Plan [Internet]. International Livestock Research Institute and UN FAO; 2017 Aug. Available from: http://www.fao.org/faolex/results/details/en/c/LEX-FAOC172923/#:~:text=The%20Rwanda%20Livestock%20Master%20Plan,%2F18%2D2021%2F22.&text=Further%2C%20the%20document%20aims%20to,and%20in%20livestock%20keeping%20households

24. Hartman A. Rift Valley Fever. Clin Lab Med. 2017 Jun;37(2):285–301.

25. Mutua EN, Bukachi SA, Bett BK, Estambale BA, Nyamongo IK. “We do not bury dead livestock like human beings”: Community behaviors and risk of Rift Valley Fever virus infection in Baringo County, Kenya. PLOS Neglected Tropical Diseases. 2017 May 24;11(5):e0005582.

26. Nyabinwa P, Kashongwe OB, Hirwa C d’Andre, Bebe BO. Perception of farmers about endometritis prevention and control measures for zero-grazed dairy cows on smallholder farms in Rwanda. BMC Veterinary Research. 2020 Jun 5;16(1):175.

27. Abdi IH, Affognon HD, Wanjoya AK, Onyango-Ouma W, Sang R. Knowledge, Attitudes and Practices (KAP) on Rift Valley Fever among Pastoralist Communities of Ijara District, North Eastern Kenya. PLoS Negl Trop Dis [Internet]. 2015 Nov 13 [cited 2020 Sep 15];9(11). Available from: https://www.ncbi.nlm.nih.gov/pmc/articles/PMC4643900/

28. Msimang V, Thompson PN, Jansen van Vuren P, Tempia S, Cordel C, Kgaladi J, et al. Rift Valley Fever Virus Exposure amongst Farmers, Farm Workers, and Veterinary Professionals in Central South Africa. Viruses. 2019 Feb 7;11(2):140.

29. Archer BN, Weyer J, Paweska J, Nkosi D, Leman P, Tint KS, et al. Outbreak of Rift Valley fever affecting veterinarians and farmers in South Africa, 2008. South African Medical Journal. 2011 Jan 1;101(4):263-66.
30. Ferrer R, Klein WM. Risk perceptions and health behavior. Curr Opin Psychol. 2015 Oct 1;5:85–9.
31. Mweya CN, Mboera LE, Kimera SI. Climate influence on emerging risk areas for Rift Valley fever epidemics in Tanzania. The American journal of tropical medicine and hygiene. 2017 Jul 12;97(1):109-14.
32. National Institute of Statistics of Rwanda. EICV3 District Profile, East-Nyagatare. Republic of Rwanda; 2011.
33. Nyagatare (District, Rwanda) - Population Statistics, Charts, Map and Location [Internet]. [cited 2020 Sep 22]. Available from: https://www.citypopulation.de/php/rwanda-sector-admin.php?adm1id=52
34. Hasanov E, Zeynalova S, Geleishvili M, Maes E, Tongren E, Marshall E, et al. Assessing the impact of public education on a preventable zoonotic disease: rabies. Epidemiol Infect. 2018 Jan;146(2):227-35.
35. Welburn SC, Beange I, Ducrotoy MJ, Okello AL. The neglected zoonoses--the case for integrated control and advocacy. Clin Microbiol Infect. 2015 May;21(5):433–43.
36. Lin L, Savoia E, Agboola F, Viswanath K. What have we learned about communication inequalities during the H1N1 pandemic: a systematic review of the literature. BMC Public Health. 2014 May 21;14:484.
37. de St. Maurice A, Nyakarahuka L, Purpura L, Ervin E, Tumusiime A, Balinandi S, et al. Rift Valley Fever: A survey of knowledge, attitudes, and practice of slaughterhouse workers and

community members in Kabale District, Uganda. PLoS Negl Trop Dis [Internet]. 2018 Mar 5 [cited 2020 Sep 15];12(3). Available from: https://www.ncbi.nlm.nih.gov/pmc/articles/PMC5860784/

38. Rich KM, Wanyoike F. An assessment of the regional and national socio-economic impacts of the 2007 Rift Valley fever outbreak in Kenya. Am J Trop Med Hyg. 2010 Aug;83(2 Suppl):52–7.

39. Songane M. Challenges for nationwide vaccine delivery in African countries. Int J Health Econ Manag. 2018 Jun 1;18(2):197–219.

40. Mihigo R, Okeibunor J, Anya B, Mkanda P, Zawaira F. Challenges of immunization in the African Region. Pan Afr Med J [Internet]. 2017 Jun 21 [cited 2021 Feb 27];27(Suppl 3). Available from: https://www.ncbi.nlm.nih.gov/pmc/articles/PMC5745929/

41. USAID Gender Analysis. Gender Analysis for USAID/Rwanda Feed the Future Project [Internet]. 2015 Dec. Available from: https://www.usaid.gov/sites/default/files/documents/1860/GA%20-%20FtF%20-%20FINAL%20December%2014%202015%20-%20Public%20Version.pdf

42. Republic of Rwanda Gender Monitoring Office. Gender and Agriculture. Kigali; 2017 Mar.

43. Muga GO, Onyango-Ouma W, Sang R, Affognon H. Sociocultural and Economic Dimensions of Rift Valley Fever. Am J Trop Med Hyg. 2015 Apr 1;92(4):730–8.

44. Mutua E, de Haan N, Tumusiime D, Jost C, Bett B. A Qualitative Study on Gendered Barriers to Livestock Vaccine Uptake in Kenya and Uganda and Their Implications on Rift Valley Fever Control. Vaccines. 2019 Sep;7(3):86.

45. John Curry. Gender and Livestock in African Production Systems: An Introduction. Human ecology : an interdisciplinary journal. 1996 Jun;24(2):149–60.

46. Mupawaenda AC, Chawatama S, Muvavarirwa P. Gender issues in livestock production: a case study of Zimbabwe. Trop Anim Health Prod. 2009 Oct;41(7):1017–21.

47. Agricultural transformation in Rwanda: Can Gendered Market Participation Explain the Persistence of Subsistence Farming? || Rivera Publications || Gender and Women's Studies [Internet]. [cited 2020 Nov 7]. Available from: http://riverapublications.com/article/agricultural-transformation-in-rwanda-can-gendered-market-participation-explain-the-persistence-of-subsistence-farming

48. Quisumbing AR, Rubin D, Manfre C, Waithanji E, van den Bold M, Olney D, et al. Gender, assets, and market-oriented agriculture: learning from high-value crop and livestock projects in Africa and Asia. Agriculture and Human Values; Dordrecht. 2015 Dec;32(4):705–25.

49. Eugene M. Characterization of Cattle Production Systems in Nyagatare District of Eastern Province, Rwanda. Rheology: Open Access. 2017 May 29;1(2):1–21.

50. National Institute of Statistics of Rwanda. The Fifth Integrated Household Living Conditions Survey EICV5 2016/2017 Main Indicators Report. 2018 Nov.

51. Birachi E, Hansen J, Radeny M, Mutua M, Mbugua M, Munyangeri Y, et al. Rwanda Climate Services for Agriculture: Evaluation of farmers' awareness, use and impacts Working Paper No. 304 CGIAR Research Program on Climate Change, Agriculture and Food Security (CCAFS). 2020.

52. Media/Psychological Impact on Multiple Outbreaks of Emerging Infectious Diseases [Internet]. [cited 2020 Nov 15]. Available from: https://www.hindawi.com/journals/cmmm/2007/612372/

53. Tchuenche JM, Bauch CT. Dynamics of an Infectious Disease Where Media Coverage Influences Transmission [Internet]. Vol. 2012, ISRN Biomathematics. Hindawi; 2012 [cited

2020 Nov 15]. p. e581274. Available from: https://www.hindawi.com/journals/isrn/2012/581274/

54. Oparinde A, Birol E, Murekezi A, Katsvairo L, Diressie MT, Nkundimana J d'amour, et al. Radio Messaging Frequency, Information Framing, and Consumer Willingness to Pay for Biofortified Iron Beans: Evidence from Revealed Preference Elicitation in Rural Rwanda. Canadian Journal of Agricultural Economics/Revue canadienne d'agroeconomie. 2016;64(4):613–52.

55. Shelus V, VanEnk L, Giuffrida M, Jansen S, Connolly S, Mukabatsinda M, et al. Understanding your body matters: Effects of an entertainment-education serial radio drama on fertility awareness in Rwanda. Journal of Health Communication. 2018 Aug 3;23(8):761–72.

56. Grace D, Randolph T, Affognon H, Dramane D, Diall O, Clausen P-H. Characterisation and validation of farmers' knowledge and practice of cattle trypanosomiasis management in the cotton zone of West Africa. Acta Tropica. 2009 Aug 1;111(2):137–43.

57. Kimani T, Schelling E, Bett B, Ngigi M, Randolph T, Fuhrimann S. Public Health Benefits from Livestock Rift Valley Fever Control: A Simulation of Two Epidemics in Kenya. Ecohealth. 2016;13(4):729–42.

Figure

Click here to access/download;Figure;Figure 1_Smith et al. RVF Knowledge Communication

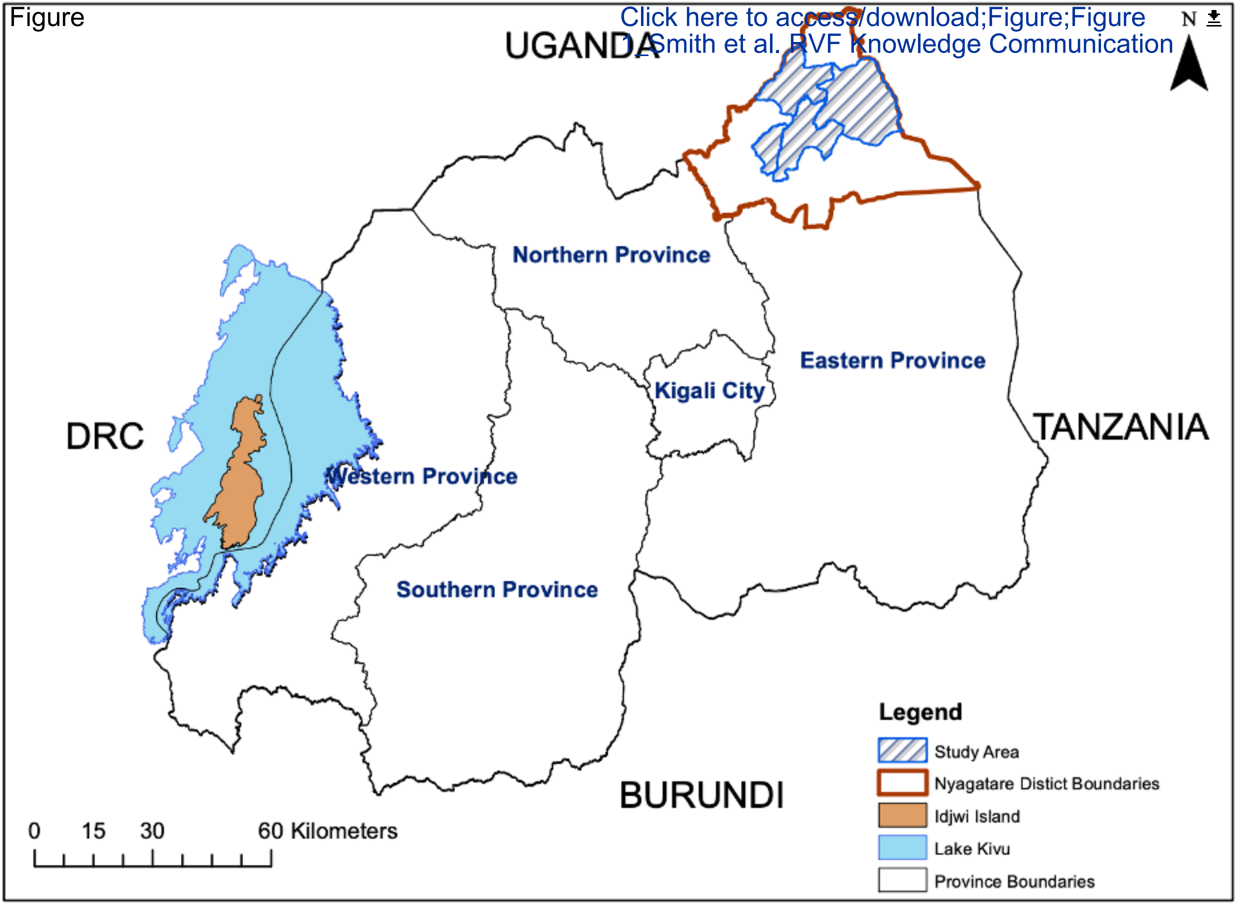

Supplement: Attachment — Submitted filename: PNTD-D-21-00602_reviewer.pdf [file pntd.0009705.s004.pdf]
