# Supplementary material for: Rift Valley fever knowledge, mitigation strategies and communication preferences among male and female livestock farmers in Eastern Province, Rwanda
Source: PLoS Negl Trop Dis. 2021 Aug 23;15(8):e0009705. doi: 10.1371/journal.pntd.0009705 (PMC8412303; doi:10.1371/journal.pntd.0009705)
Supplement: Attachment — Submitted filename: PNTD Reviewer.docx [file pntd.0009705.s002.docx]

**PNTD Reviewer Comment**

**Comments to the Author**
This study provides good basic background data important for improving understanding of Rift Valley fever (RVF) occurrence. The scope and extent of the study is applaudable. However, there are few shortcomings in the manuscript and must be addressed.
**Introduction –** This is too long. It should be summarized to two to two and half pages, covering background, statement of the problems, justifications, and objectives. As it is, some of the narrations are important but not required.

**Materials and methods –** As it is currently written, the study design is understandable, but the analytical method is difficult to follow. Why the use Fisher’s exact test and not Chi-square test in a situation where data in boxes is more than five? Please provide reference when responding to this question. If a revision is undertaken I suggest being very clear about each step of the analysis. Take care to specifically state the determinants and outcomes evaluated. It is surprising that you are dealing with associations and didn’t take care of confounders and effect modifiers. Why?
**Results –** Please rearrange your independent and dependent variables in Tables 3 - 5. Can gender be dependent variable? Please, justify with references
**Discussion –** The contents of this section is too lengthy with some not very important observations. Please revise adequately.

Lines 433-434: “First, our study population was relatively small. Analyses were done with a Fisher’s Exact Test to accommodate the small sample size.” What do you mean by this statement and substantiate with references?
**Conclusions and Recommendations –** This section did not give any conclusion on the findings of the study, but did provide only recommendations. Please give conclusions on the various findings.

Move lines 442-443 “Gendered approaches to radio-based information dissemination should be considered, as radio has found to be preferred and effective [35, 38]” to the ‘Discussion’ section. References are not required here.

**Others –** The questionnaire is missing – should be provided as supplementary material.
Thanks for all your hard work to improve our understanding of this important topic!
