# Supplementary material for: Rift Valley fever knowledge, mitigation strategies and communication preferences among male and female livestock farmers in Eastern Province, Rwanda
Source: PLoS Negl Trop Dis. 2021 Aug 23;15(8):e0009705. doi: 10.1371/journal.pntd.0009705 (PMC8412303; doi:10.1371/journal.pntd.0009705)
Supplement: Attachment — Submitted filename: Reviewer_PNTD-D-21-00602_RVF_Rwanda.docx [file pntd.0009705.s003.docx]

**Reviewer's report**

**Title:** A survey of knowledge, risk perceptions, mitigation strategies, and gender-nuanced communication practices among livestock farmers in Eastern Province following the first officially declared Rift Valley Fever outbreak in Rwanda

**Date:** 14 May 2021

**Review of Manuscript**

**Summary**: In many parts of Africa, a lot of people earn their living by keeping ruminants and for that reason, animal diseases could have a direct impact on the sources of income but also on the general population that could not have longer access to animal protein, leading to malnutrition for the poorest. RVF remains to be a threat to livestock keepers and nations where the disease is occurring due to its major economic implications through the costs of the measures taken at individual, collective and international levels in order to prevent or control infection and disease outbreaks.

The objectives of this study were to evaluate knowledge and risk perceptions of RVF transmission among livestock farmers in Nyagatare District, Eastern Province, Rwanda, and to compare RVF knowledge, risk perceptions, and farming practices between male and female livestock farmers. Data were collected using questionnaires from livestock owners in the four sectors of Nyagatare District in the Eastern Province of Rwanda in June 2019. Results highlight that there was little knowledge on disease.

Generally, information regarding the awareness, atitude and practices towards controlling RVF from other countries is available in the literature. However, it is advisable that each individual country should assess the level of knowledge and mitigation strategies for RVF. This information remain scarce and such data could be useful in the future to better address the challenges of RVF outbreaks in Rwanda. In conclusion, I consider this article as very interesting and could be of interest for broad range of readers especially within sub–Saharan Africa, Middle East, and countries at risk. However, there are some minor revisions that need to be addressed before being considered for publication. Apart from this remark, the interpretation and conclusion are for the most part sound, justified by the data and consistent with the objectives.

**Specific concerns**

**Introduction**

1. A citation is need at line 99 as indicated in the manuscript
2. What is the implication (s) of the detected antibodies in 2012 in Rwanda? Is there a link between the detected antibodies in 2012 and the occurrence of RVF outbreak in 2018? -line 101 and 102
3. When was the animals vaccinated? Were the animals where outbreak occurred also vaccinated? What type of vaccine did they use? -line 113 and 114

**Methods**

**Description of the study area**

1. The four study sectors of Nyagatare district in the Eastern Province should be identified in the map by either numbers (1-4) or any other identification and described in the figure legends. The Island, Lake and province boundaries in the legends can be removed and replaced with the study sectors.

**Study design and data collection**

1. In this study they used questionnaire tool to collect data and it is not known as to why other tools such as in-depth interviews with key informants (veterinarians, official leaders, and field officers) involved in control of RVF and focal groups were not used in this study to get more information on the study objectives
2. The sentence in line 203 – 207 is not clear, consider rephrasing it.
3. Which participants are you referring to, at the three milk collection centres and the two village meeting points or the door-to-door visits? Please rephrase or clarify -line 207-209.
4. The research team claim to have sampled exhaustively in the selected sampling areas including the door-to-door visits (line 203 – 207). The sampled livestock farmers (123 farmers) in this study, are they the only livestock keepers in the four sectors? What was the criteria for selecting the sampling approach (sampling locations)?
5. Did any member in the family where livestock were kept had equal chance of being included if he/she was above 18 years old and available? If you found a family where both male and female were present how did you decide to include the gender? -line 208 and 209
6. Please indicate how many people/households were involved in the pre-testing – line 220.
7. Who did collect the data; were they trained to do it?

**Results**

1. How was the gender taken into consideration during sample selection/administration of the questionnaire?
2. In table 2, it shows that only one person/household from Rukomo sector and 13 from Nyagatare sector out of 123 livestock farmers were administered the questionnaire. What criteria did the authors use to divide the questionnaires among the four sectors?
3. Those who were not likely to call a veterinary doctor when their animals got sick, how did they do with the sick animal(s)? -line 304 and 305

**Discussion**

1. The answers as to why some livestock farmers did not vaccinate their animals despite of the past vaccination campaigns could be obtained through open ended questions, in-depth interviews with key informants or focus group discussions. But it is not known why this was not done in the current study -line 344 – 350
2. Were cattle owners or their children/workers taking cattle for vaccinations? Do you have any clue on how the information was taken back to all the family members who had chance to be included in your study? -line 419 and 420
3. Was the radio accessible by the females in Rwanda? -line 425
4. One of the limitations in this study was the small sample size. What criteria did you use to obtain your sample size?

**Conclusion**

In conclusion, all this comments and those inserted in the manuscript are considered minor and the paper may be reconsidered after revision and clarification.
