# Supplementary material for: Rift Valley fever knowledge, mitigation strategies and communication preferences among male and female livestock farmers in Eastern Province, Rwanda
Source: PLoS Negl Trop Dis. 2021 Aug 23;15(8):e0009705. doi: 10.1371/journal.pntd.0009705 (PMC8412303; doi:10.1371/journal.pntd.0009705)
Supplement: Attachment — Submitted filename: Smith et al. PLOS NTD Reviewer Letter_RVF Rwanda_717.docx [file pntd.0009705.s005.docx]

Department of Infectious Disease and Global Health

Cummings School of Veterinary Medicine at Tufts University

50 Willard Street, North Grafton, MA, USA 01536

July 17, 2021

ATTN:

PLOS Neglected Tropical Diseases

San Francisco, California, USA

Re: Manuscript Revisions (PNTD-D-21-00602)

Dear Dr. Peter Hotez, Dr. Shaden Kamhawi, and Dr. Paul Brindley,

Thank you very much for the opportunity to revise and resubmit our manuscript, “Rift Valley Fever knowledge, mitigation strategies and communication preferences among male and female livestock farmers in Eastern Province, Rwanda” (PNTD-D-21-00602).

We appreciate the time that reviewers spent in reviewing our manuscript and we are grateful for their recommendations to improve the manuscript.  We responded to each of the reviewers’ comments below and amended the manuscript as requested. We have sent both a clean version of the amended manuscript as well as one with tracked changes to show where changes were made in the text. We have also sent the questionnaire used for this study.

We hope that we have revised this work to the satisfaction of all editors and reviewers, and that it can now be considered suitable for inclusion in your journal.  This research has not been published elsewhere and has not been submitted to any other journal while under consideration by PLOS-Neglected Tropical Diseases. All authors participated in the research and concur with the revised submission.

Thank you for your consideration of this manuscript and for the reviewer comments to improve it.

Sincerely,

Lindsay Smith

DVM/MA (International Relations) Candidate

Cummings School of Veterinary Medicine at Tufts University

****The line numbers referenced in blue text in these responses are the ones on the manuscript document with tracked changes. However, the tracked changes document continues to change line numbers every time the document is opened and we have not been able to fix this issue. In case there are discrepancies between the line numbers listed in this letter and in the tracked changes manuscript document, we have listed the line numbers associated with these edits in the clean version of the manuscript in red font so it can be referred to as well if needed.****

**Reviewer 1 Specific Concerns**

**Introduction**

1. A citation is need at line 99 as indicated in the manuscript

Thank you. A citation was added at line 95 in the manuscript (80 in the clean version).

2. What is the implication (s) of the detected antibodies in 2012 in Rwanda? Is there a link between the detected antibodies in 2012 and the occurrence of RVF outbreak in 2018? -line 101 and 102

Thank you for raising these questions. The implications of detected antibodies show that the virus was circulating in Rwanda prior to the first declared outbreak in 2018. Additionally, RVF vaccines were sporadically given in areas of Rwanda before 2012. Because detection of antibodies was ELISA based, the possibility of antibodies being vaccinal cannot be ruled out. We have not found a directly established link or research on this specific topic, but it is possible that there is a link between the detected antibodies in 2012 and occurrence of the RVF outbreak in 2018. According to the literature, antibodies can circulate during an inter epizootic/epidemic period. Circulating antibodies in 2012 show that RVF was likely present in Rwanda prior to the 2018 outbreak, that the animals with detected antibodies were involved in an enzootic cycle in the interepidemic period, and that animals with high enough titers were likely protected from the 2018 outbreak. The level of antibodies may also be insufficient to prevent another large outbreak with the right conditions in place which could have contributed to the 2018 outbreak. This explanation has been added in lines 95-107 (lines 80-92 in the clean version) in the manuscript using the citations here below:

a. Linda Peniel Salekwa, Philemon Nyangi Wambura, Mirende Kichuki Matiko, Douglas M. Watts. Circulation of Rift Valley Fever Virus Antibody in Cattle during Inter-Epizootic/Epidemic Periods in Selected Regions of Tanzania. The American Journal of Tropical Medicine and Hygiene. 2019 Aug;101(2):459–66.

b. Yusuf B. Ngoshe, Alida Avenant, Melinda K. Rostal, William B. Karesh, Janusz T. Paweska, Whitney Bagge, et al. Patterns of Rift Valley fever virus seropositivity in domestic ruminants in central South Africa four years after a large outbreak. Nature Scientific Reports [Internet]. 2020 Mar 26 [cited 2021 May 31];10(5489). Available from: https://www.nature.com/articles/s41598-020-62453-6#citeas

3. When was the animals vaccinated? Were the animals where outbreak occurred also vaccinated? What type of vaccine did they use? -line 113 and 114

Thank you for these questions. Vaccination campaigns occur annually in areas where RVF outbreaks have been reported. The vaccination is voluntary and farmers may be requested to pay a fee to get their cattle vaccinated. Currently, Rwanda uses a live attenuated RVF vaccine manufactured by Biopharma in Morocco. This information has been added to the manuscript at lines 95-180 (lines 93-97 in the clean version).

4. Comment in manuscript (lines 118-119): “Even without vaccination, RVF tends to end naturally and start circulating at low levels in the animals and vectors. This could be due to decreased vectors during the dry seasons.”

Thank you for this point of clarification. We now acknowledge this information in lines 180-182 (lines 97-99 in the clean version). The decreased vectors in the dry season and gradually decreasing RVF levels, combined with a vaccination campaign, likely controlled the 2018 RVF outbreak.

**Methods**

Description of the study area

1. The four study sectors of Nyagatare district in the Eastern Province should be identified in the map by either numbers (1-4) or any other identification and described in the figure legends. The Island, Lake and province boundaries in the legends can be removed and replaced with the study sectors.

Thank you for this suggestion. We have modified our map (Figure 1 at line 674) (line 170 in the clean version) accordingly.

Study design and data collection

1. In this study they used questionnaire tool to collect data and it is not known as to why other tools such as in-depth interviews with key informants (veterinarians, official leaders, and field officers) involved in control of RVF and focal groups were not used in this study to get more information on the study objectives.

Thank you for this question; this was addressed with an edit we made in lines 1186-1192 (lines 377-383 in the clean version) of the manuscript. This study was designed to assess the status of RVF knowledge and awareness specifically among livestock farmers in Eastern Province, Rwanda. To conduct open-ended, in-depth interviews or focus groups or key informant interviews with ministry officials was outside the scope of this study but we agree it is a limitation.

2. The sentence in line 203 – 207 is not clear, consider rephrasing it.

Thank you for raising this concern. We rewrote this sentence to clarify the structure of the study as well as the sample collection strategies. Please refer to lines 670-680 and 682-683 in the manuscript for these edits (175-185 and 187-188 in the clean version).

3. Which participants are you referring to, at the three milk collection centres and the two village meeting points or the door-to-door visits? Please rephrase or clarify -line 207-209.

Thank you. All participants were sampled exhaustively at all locations within the given timeframe. This has been clarified (lines 670-677 and 682-687) (175-182 and 187-192 in the clean version).

4. The research team claim to have sampled exhaustively in the selected sampling areas including the door-to-door visits (line 203 – 207). The sampled livestock farmers (123 farmers) in this study, are they the only livestock keepers in the four sectors? What was the criteria for selecting the sampling approach (sampling locations)?

Thank you for this question. A true estimate of livestock farmers within and their distribution across the four sectors is not available. The 123 livestock farmers we surveyed were only a sample of the overall population of livestock farmers across these four sectors and did not encompass the entire population of livestock farmers in those areas. Our research team maximized the probability of engaging livestock farmers in these areas by conducting our survey at milk collection centers, door-to-door visits, village meeting points, and cooperative meetings. This information is included in lines 679-682 (182-185 in the clean version) in the manuscript document. Selecting sampling locations was based on their proximity to previous sectors with recorded RVF outbreaks, total cattle production, and proximity to rice wetlands. This information was added to lines 628-633 (150-155 in the clean version) of the manuscript. Data from prior projects conducted by researchers on our team regarding the distribution of livestock farmers in Eastern Province sectors also guided our sampling location decisions.

5. Did any member in the family where livestock were kept had equal chance of being included if he/she was above 18 years old and available? If you found a family where both male and female were present how did you decide to include the gender? -line 208 and 209

Thank you for this question. We clarified this in lines 704-709 (190-192 in the clean version). The probability of sampling male/female respondents was not equal due to differences in farming and domestic activities. Those at the milk collection center were predominantly male while those at community meeting points and at home were predominantly female. If we found a family where both male and female members were present, the person primarily responsible for caretaking was selected to be interviewed for our study.

6. Please indicate how many people/households were involved in the pre-testing – line 220.

Thank you. We pre-tested our questionnaire on six volunteers. This was added to the manuscript at line 721 (203 in the clean version).

7. Who did collect the data; were they trained to do it?

Thank you. The data was collected by two veterinarians and a veterinary student who were part of the research team. They were each trained on how to collect interview data and enter it into KoboToolbox on cellphones. This was added to the manuscript from lines 724-726 (206-208 in the clean version).

**Results**

1. How was the gender taken into consideration during sample selection/administration of the questionnaire?

Thank you for this question. We added 2 sentences in the study design and data collection sentence to describe how gender was taken into account during administration of the questionnaire (lines 705-709) (lines 188-192 in the clean version).

2. In table 2, it shows that only one person/household from Rukomo sector and 13 from Nyagatare sector out of 123 livestock farmers were administered the questionnaire. What criteria did the authors use to divide the questionnaires among the four sectors?

Thank you for asking this question. Our team targeted livestock farmers at various sampling locations, including milk collection centers, village meeting points, door-to-door visits on homesteads in Rwimiyaga and Rwempasha. The majority of livestock farmers who were interviewed reported being from Rwimiyaga and Rwempasha. At the milk collection centers in these sectors, a small number of livestock farmers met our inclusion criteria but were from Nyagatare and Rukomo sectors. These farmers lived in Nyagatare and Rukomo sectors and either worked in or delivered milk to milk collection centers in Rwempasha and Rwimiyaga, and we recorded the sector in which they lived within our dataset. If livestock farmers were present at our sampling locations and fit the criteria of livestock ownership or being involved in livestock-related activities, they were included in our study. A clarifying caption with this information was added under Table 2 in the manuscript in lines 763-765 (229-231 in the clean version).

3. Those who were not likely to call a veterinary doctor when their animals got sick, how did they do with the sick animal(s)? -line 304 and 305

Thank you for this question. Respondents who reported not calling a veterinarian when their animals got sick reported purchasing tetracycline at a pharmacy and treating their animals with this antibiotic themselves. This was added to the manuscript at lines 831-832 (288-289 in the clean version) to clarify this.

**Discussion**

1. The answers as to why some livestock farmers did not vaccinate their animals despite of the past vaccination campaigns could be obtained through open ended questions, in-depth interviews with key informants or focus group discussions. But it is not known why this was not done in the current study -line 344 – 350

Thank you for raising this concern. As stated above, the goals of this pilot study were to collect a general baseline level of information about RVF knowledge, risk perceptions of RVF, communication and information sharing strategies, and zoonosis risk mitigation strategies among male and female livestock farmers in Eastern Province. We agree that additional information from respondents would help to better understand vaccine hesitancy in livestock. However, it is outside the scope of this research. While we did not conduct key informant interviews, we did ask some open ended questions to farmers within our survey interviews. We have addressed this in an addition we made from lines 1188-1194 (lines 377-383 in the clean version) in the manuscript.

2. Were cattle owners or their children/workers taking cattle for vaccinations? Do you have any clue on how the information was taken back to all the family members who had chance to be included in your study? -line 419 and 420

Thank you for asking this question. This would be very useful information in determining how information is discussed and spreads through communities. Our team unfortunately did not ask about how information was spread or taken back to other family members, but we encourage this being explored in future studies to determine how information spreads following campaigns. This was added in lines 1191-1197 (lines 383-389 in the clean version) in the manuscript. While we did not ask about how information was taken back to family members specifically, we did ask interviewees about trusted sources of information and who they viewed as credible. This provided some information about how information is disseminated through communities.

3. Was the radio accessible by the females in Rwanda? -line 425

Thank you for this question. We clarified that it is accessible to both male and female livestock farmers with an edit in lines 1063-1064 (lines 366-367 in the clean version). Many female livestock farmers reported thinking that radio and talking on the telephone were the best ways to receive information.

4. One of the limitations in this study was the small sample size. What criteria did you use to obtain your sample size?

Thank you for this question. Our total sample size was a convenience sample. The size and scope of our convenience sample was influenced by time and budget. This detail was added at lines 693-699 (176-182 in the clean version) of the manuscript. This pilot study should be replicated in the future with more robust, probabilistic sampling methods.

**Reviewer 2 Specific Concerns**

**Introduction**

1. This is too long. It should be summarized to two to two and half pages, covering background, statement of the problems, justifications, and objectives. As it is, some of the narrations are important but not required.

Thank you for this comment. The introduction has been significantly shortened down to 2.5 pages with tracked changes to highlight changes made. References were deleted with the deleted text and subsequently re-ordered as well.

**Materials and methods**

1. As it is currently written, the study design is understandable, but the analytical method is difficult to follow. Why the use Fisher’s exact test and not Chi-square test in a situation where data in boxes is more than five? Please provide reference when responding to this question. If a revision is undertaken I suggest being very clear about each step of the analysis. Take care to specifically state the determinants and outcomes evaluated. It is surprising that you are dealing with associations and didn’t take care of confounders and effect modifiers. Why?

Thank you for these questions and suggestions. We used a Fisher’s Exact test because of our small sample size of 123 farmers. The Fisher’s Exact Test is more accurate for small sample sizes of less than 500 than the Pearson Chi-squared and is the ideal statistical test for small sample sizes. These two citations support these points and they were added at lines 1169-1170 (373-374 in the clean version) in the manuscript.

References:

a. McDonald JH. Handbook of biological statistics. Baltimore, MD: sparky house publishing; 2009 Aug. 70-74.
b. Kim H-Y. Statistical notes for clinical researchers: Chi-squared test and Fisher’s exact test. Restor Dent Endod. 2017 May;42(2):152–5.

Additionally, categorical data, qualitative data and dichotomous data do not follow the normal distribution. We have two groups (male and female) with more than 2 data sets. For our study we are looking for differences between males and females in risk perceptions regarding RVF which is a comparison. Gender was the main determinant that we evaluated and the outcomes were knowledge about RVF, risk perceptions of RVF, risk mitigation strategies, preferred sources of information and communication practices, and attitudes toward sources of information as displayed in Tables 3-6.

Regarding effect modifiers, confounders, and outliers, we did not control for these as we did not perform multivariate regression. This analysis fell out of the scope of our study which was solely focused on gathering baseline survey data as a pilot study.

**Results**

1. Please rearrange your independent and dependent variables in Tables 3 - 5. Can gender be dependent variable? Please, justify with references.

Thank you for asking about this. Gender is the independent variable in Tables 3-5 and is in the vertical columns, and the dependent variables are interview question responses. We decided to put gender in the columns as the independent variable for display purposes and clarity, and it is acceptable to display it in vertical columns within tables to analyze the influence that these variables have on variables displayed in the rows.

Reference: Bertani A, Di Paola G, Russo E, Tuzzolino F. How to describe bivariate data. J Thorac Dis. 2018 Feb;10(2):1133–7.

**Discussion**

1. The contents of this section is too lengthy with some not very important observations. Please revise adequately.

Thank you for this comment. The discussion was shortened with tracked changes to highlight changes made. References were deleted with the deleted text and subsequently re-ordered as well.

2. Lines 433-434: “First, our study population was relatively small. Analyses were done with a Fisher’s Exact Test to accommodate the small sample size.” What do you mean by this statement and substantiate with references?

Thank you for this question. We agree that this was a confusing statement that was unclear and we revised it. The Fisher’s Exact Test did not “accommodate the small sample size” and did not “solve” the issue of having a small sample size. Rather, it is merely the more appropriate test to be used in analyzing small sample sizes and we chose it to analyze our small dataset (references below). We added a sentence stating that our sample size was relatively small, and that this was a pilot study that should be scaled up to a larger sample size and replicated in the future. This new sentence can be found from lines 1169-1170 (373-375 in the clean version) of the manuscript.

References with information about using the Fisher’s Exact Test for small sample sizes:

a. McDonald JH. Handbook of biological statistics. Baltimore, MD: sparky house publishing; 2009 Aug. 70-74.
b. Kim H-Y. Statistical notes for clinical researchers: Chi-squared test and Fisher’s exact test. Restor Dent Endod. 2017 May;42(2):152–5.

**Conclusions and Recommendations**

1. This section did not give any conclusion on the findings of the study, but did provide only recommendations. Please give conclusions on the various findings.

Thank you for this comment. We included a concluding statement as part of the discussion. It has been moved to a standalone section from lines 1188-1201 (392-395 in the clean version) in the manuscript.

2. Move lines 442-443 “Gendered approaches to radio-based information dissemination should be considered, as radio has found to be preferred and effective [35, 38]” to the ‘Discussion’ section. References are not required here.

Thank you for this suggestion. We moved this sentence up to the discussion in lines 1037-1040 (355-358 of the clean version) of the manuscript and removed the references.

**Others**

1. The questionnaire is missing – should be provided as supplementary material.
   Thanks for all your hard work to improve our understanding of this important topic!

Thank you so much for appreciating this topic. We have attached the questionnaire as supplementary material and have included it in our submission.
