# Supplementary material for: Rift Valley fever knowledge, mitigation strategies and communication preferences among male and female livestock farmers in Eastern Province, Rwanda
Source: PLoS Negl Trop Dis. 2021 Aug 23;15(8):e0009705. doi: 10.1371/journal.pntd.0009705 (PMC8412303; doi:10.1371/journal.pntd.0009705)
Supplement: Attachment — Submitted filename: Reviewer_PNTD-D-21-00602_RVF_Rwanda-2.docx [file pntd.0009705.s006.docx]

**Reviewer's report**

**Title:** Rift Valley fever knowledge, mitigation strategies and communication preferences among male and female livestock farmers in Eastern Province, Rwanda

**Date:** 04 August 2021

**Review of the revised Manuscript**

The subject falls within the scope of the journals.

The author (s) have tried to answer and respond to the numbers of the issues or concern raised based on the previous review. I declare that I am satisfied with their responses on the queries I raised in the manuscript.
